# Genome-Wide Estimates of Coancestry, Inbreeding and Effective Population Size in the Spanish Holstein Population

**DOI:** 10.1371/journal.pone.0124157

**Published:** 2015-04-16

**Authors:** Silvia Teresa Rodríguez-Ramilo, Jesús Fernández, Miguel Angel Toro, Delfino Hernández, Beatriz Villanueva

**Affiliations:** 1 Departamento de Mejora Genética Animal, INIA, Madrid, Spain; 2 Departamento de Producción Animal, Escuela Técnica Superior de Ingenieros Agrónomos, Madrid, Spain; 3 Departamento Técnico Conafe, Madrid, Spain; University of Florida, UNITED STATES

## Abstract

Estimates of effective population size in the Holstein cattle breed have usually been low despite the large number of animals that constitute this breed. Effective population size is inversely related to the rates at which coancestry and inbreeding increase and these rates have been high as a consequence of intense and accurate selection. Traditionally, coancestry and inbreeding coefficients have been calculated from pedigree data. However, the development of genome-wide single nucleotide polymorphisms has increased the interest of calculating these coefficients from molecular data in order to improve their accuracy. In this study, genomic estimates of coancestry, inbreeding and effective population size were obtained in the Spanish Holstein population and then compared with pedigree-based estimates. A total of 11,135 animals genotyped with the Illumina BovineSNP50 BeadChip were available for the study. After applying filtering criteria, the final genomic dataset included 36,693 autosomal SNPs and 10,569 animals. Pedigree data from those genotyped animals included 31,203 animals. These individuals represented only the last five generations in order to homogenise the amount of pedigree information across animals. Genomic estimates of coancestry and inbreeding were obtained from identity by descent segments (coancestry) or runs of homozygosity (inbreeding). The results indicate that the percentage of variance of pedigree-based coancestry estimates explained by genomic coancestry estimates was higher than that for inbreeding. Estimates of effective population size obtained from genome-wide and pedigree information were consistent and ranged from about 66 to 79. These low values emphasize the need of controlling the rate of increase of coancestry and inbreeding in Holstein selection programmes.

## Introduction

It is well known that the effective size of a population (*N*_*e*_) is usually less than its census size as there are often deviations from the assumptions of the idealized population by having unequal sex ratios, variation in family size, unequal numbers in successive generations and overlapping generations [1]. A clear example of this is given by the Holstein cattle breed that despite being numerically very large (millions of animals spread across the world), shows a *N*_*e*_ of the order of 100 [2, 3] when calculated from the rate of inbreeding (Δ*F*) computed from pedigree data.

Holstein dairy cattle have dominated the milk production industry over decades. Intense and accurate artificial selection practised over many years has resulted in high rates of genetic gain for milk production traits. This has implied an extreme use of a limited number of elite sires of high genetic merit for production traits via artificial insemination. However, the high rates of gain have been accompanied by large increases in the rates at which inbreeding and coancestry (Δ*f*) accumulate and, consequently, by large reductions in *N*_*e*_. This has led, in the last decades, to an increasing awareness of the need of measuring and controlling the loss of genetic variation and the increase in inbreeding to mitigate its negative effects [4]. These include inbreeding depression in production traits and reproductive ability [5, 6, 7, 8] and an increase in the prevalence of undesirable genetic disorders such as the complex vertebral malformation (CVM) [9], deficiency of uridine monophosphate synthase (DUMPS) [10], brachyspina syndrome (BS) [11] and Bovine leucocyte adhesion deficiency (BLAD) [12].

With the advances in high-throughput genotyping techniques and the development of chips containing thousands of SNPs at a reasonable cost, the implementation of genome-wide evaluation [13] has become routine in many large scale commercial Holstein breeding programmes [14, 15]. As high levels of accuracy can be achieved at an early age, generation intervals can be shortened leading to faster gain [16]. Although genomic selection leads to decreased rates of inbreeding per generation in comparison with BLUP selection [17, 18, 19], it is not inbreeding free. Thus, the need for measuring and controlling inbreeding remains [17].

Traditionally, coancestry and inbreeding coefficients have been estimated from pedigree records. However, the dense marker chips that have been developed for cattle with the main purpose of increasing selection responses, can be also used for obtaining genome-wide estimates of coancestry and inbreeding. Genomic estimates are expected to be more accurate than pedigree-based estimates because they i) reflect the actual percentage of the genome that is homozygous (inbreeding) or the actual percentage of the genome shared by two individuals (coancestry) whereas pedigree-based estimates are only expectations of such percentages; and ii) are able to capture relationships due to very distant common ancestors that pedigree-based estimates ignore [20].

In livestock species, genomic estimates of inbreeding have been obtained for different cattle [8, 21, 22, 23, 24], sheep [25] and pig [26, 27] breeds, but estimates of genomic coancestry are rare [28]. However, both inbreeding and coancestry are of fundamental importance in animal breeding programmes. Although inbreeding depression depends on the levels of inbreeding and not on coancestry, in scenarios with non-random mating such as those in most cattle breeding programmes, the rate at which genetic variability is lost is better measured by Δ*f* than by Δ*F* [29].

The objective of this study was to obtain genomic estimates of coancestry and inbreeding coefficients and *N*_*e*_ in the Spanish Holstein population. Genomic estimates were then compared to those based on pedigree information.

## Materials and Methods

### Genomic and pedigree data

**Fig 1:**
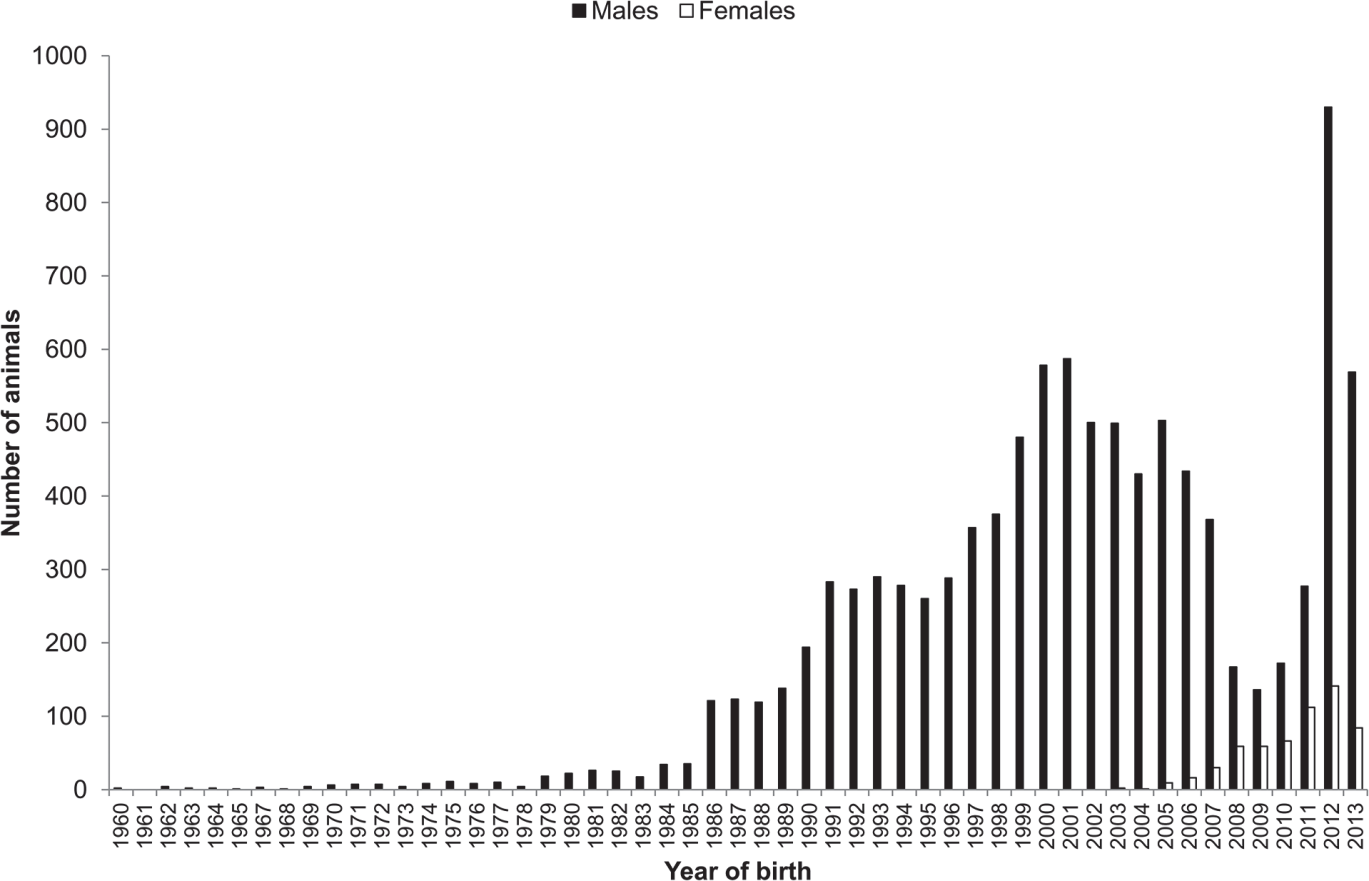
Distribution of genotyped animals by year of birth.

Genomic information from 11,135 animals belonging to the Spanish Holstein population was analyzed in this study. These individuals were genotyped with the Illumina BovineSNP50 BeadChip (versions v1 or v2). Only SNPs common to both chip versions were selected for the analysis (52,340 SNPs). SNPs positions within the genome were obtained from the UMD 3.0 bovine genome assembly [30]. Unmapped SNPs (523) and those mapped on chromosomes X or Y (1,056) were excluded. In addition, 14,068 SNPs with missing genotypes for more than 5% of the individuals were discarded. After that, 566 animals with more than 5% missing genotypes for the remaining 36,693 SNPs were also removed. The final dataset included 36,693 autosomal SNPs and 10,569 animals (9,990 bulls and 579 cows). The distribution of genotyped animals by year of birth is shown in Fig 1.

The pedigree data set, provided by the Spanish Holstein Association (Conafe), was constructed with all known ancestors of genotyped individuals and comprised 35,473 animals. The software Endog [31] was used to calculate the average numbers of generations traced (14.50), full traced generations (2.56) and equivalent complete generations (6.12). The generation interval (*L*) was calculated as the average age of parents when their offspring was born. The average generation intervals for sires of bulls, dams of bulls, sires of cows and dams of cows were 6.06, 3.88, 5.24 and 3.77, respectively. The average weighted *L* across paths and years was 4.2 years. We observed that *L* has decreased across time. The average *L* for the periods 1960 to 1979, 1980 to 1999 and 2000 to 2013 were 5.1, 4.2 and 3.3, respectively.

### Genomic estimates of inbreeding and coancestry

Genomic estimates of inbreeding coefficients were obtained from runs of homozygosity (ROHs) which are long, uninterrupted stretches of homozygous genotypes [20]. Specifically, the inbreeding estimator, *F*_*ROH*_, is the proportion of the genome that is in ROH. For individual *i*, $F_{ROH_{i}}$ was calculated as

$$F_{ROH_{i}}=\frac{\sum_{k=1}^{n_{ROH_{i}}}l_{ROH_{ik}}}{l_{g}},$$

where $n_{ROH_{i}}$ is the total number of ROHs in individual *i*, $l_{ROH_{ik}}$ is the length of the *k* ^*th*^ ROH in individual *i* in base pairs and *l*_*g*_ is the total length of the genome in base pairs.

The criteria used for defining a ROH were as follows: (i) the minimum length that constituted a ROH was 4 Mb; (ii) the minimum number of SNPs was 30; (iii) the minimum density was 1 SNP per 100 kb; (iv) the maximum distance allowed between two consecutive homozygous SNPs in a run was 1 Mb; and (v) a maximum of two missing genotypes and one heterozygous genotype within a particular ROH were permitted. The choice of 4 Mb for the minimum length permitted for defining a ROH was based on the results of Ferenčaković et al. [26] who showed that *F*_*ROH*_ based on shorter segments systematically overestimates autozygosity when using the Illumina BovineSNP50 BeadChip.

The coancestry coefficient based on IBD segments (*f*_*SEG*_) between individuals *i* and *j* was calculated as

$$f_{SEG_{ij}}=\frac{\sum_{k=1}^{n_{SEG_{ij}}}\sum_{a_{i}=1}^{2}\sum_{b_{j}=1}^{2}l_{SEG_{k}}(a_{i},b_{j})}{4l_{g}},$$

where $l_{SEG_{k}}(a_{i},b_{j})$ is the length of the *k* ^*th*^ shared IBD segment measured over homologue *a* of individual *i* and homologue *b* of individual *j* and $n_{SEG}{}_{ij}$ is the total number of IBD segments between individuals *i* and *j*. The criteria for defining an IBD segment were equivalent to those used for defining a ROH. Both $F_{ROH_{i}}$ and $f_{SEG_{ij}}$ were obtained using the software developed by de Cara et al. [32].

In order to calculate coancestry coefficients based on IBD segments, haplotypes need to be inferred from genotype data. The phase inference was performed using the software BEAGLE [33] that uses localized haplotype clustering and fits the data through an Expectation-Maximization (EM) algorithm. Each chromosome was phased independently using the default parameters (10 iterations). After haplotype inference, $f_{SEG_{ij}}$ was obtained as explained above.

### Genomic estimates of homozygosity and similarity

We also obtained genomic estimates of molecular homozygosity and molecular similarity on a SNP-by-SNP basis. The genomic similarity between individuals was obtained by applying Malécot’s definition [34] to the marker loci; that is, the molecular similarity between individuals *i* and *j* ($S_{SNP_{ij}}$ **)** is the probability that two alleles at a given locus taken at random from each individual are equal (IBS). Analogously, the genomic homozygosity of individual *i* ($H_{SNP_{i}}$) is the probability that the two alleles carried by this individual at a given locus are IBS. In this study, $S_{SNP_{ij}}$ was calculated as

$$S_{SNP}{}_{{}_{ij}}=(1/n_{s})\sum_{s=1}^{n_{s}}\left[\left(\sum_{k=1}^{2}\sum_{m=1}^{2}I_{s_{k_{i}m_{j}}}\right)/4\right],$$

where *n*_*s*_ is the number of SNPs and $I_{s_{k_{i}m_{j}}}$ is the identity of the *k* ^*th*^ allele from individual *i* with the *m* ^*th*^ allele from individual *j* at SNP *s*, and takes a value of 1 if both alleles are identical and 0 otherwise. The genomic homozygosity of individual *i* was calculated as $H_{SNP_{i}}=2S_{SNP_{ii}}-1$, and was equal to the proportion of homozygous SNPs.

### Pedigree-based estimates

The pedigree-based coancestry ($f_{PED_{ij}}$) and inbreeding ($F_{PED_{i}}$) coefficients for the genotyped individuals were calculated by going back only five generations in the pedigree in order to homogenise the amount of pedigree information across individuals. This data set included 31,203 animals. Estimates of both coefficients were obtained using the software PEDIG [35] with the option that implements the algorithm of Meuwissen and Luo [36].

### Rates of change in coancestry and inbreeding, and effective population size

Rates of change in genomic and pedigree-based inbreeding per year (Δ*F*_*ROH*(*y*)_ and Δ*F*_*PED*(*y*)_, respectively) were computed by regressing the natural logarithm of (1—*F*) for each individual on the year of birth. The slopes of these regressions are approximately equal to –Δ*F*_*ROH*(*y*)_ and –Δ*F*_*PED*(*y*)_. Rates of change in inbreeding per generation (Δ*F*_*ROH*_ and Δ*F*_*PED*_) were calculated by multiplying the rates per year by the generation interval. Finally, estimates of *N*_*e*_ were obtained from the rate of change in inbreeding per generation as *N*_*eF_ROH*_ = 1/2Δ*F*_*ROH*_ and *N*_*eF_PED*_ = 1/2Δ*F*_*PED*_. Rates of change in coancestry per year (Δ*f*_*SEG*(*y*)_ and Δ*f*_*PED*(*y*)_), and per generation (Δ*f*_*SEG*_ and Δ*f*_*PED*_), and effective population size (*N*_*ef_SEG*_ = 1/2Δ*f*_*SEG*_ and *N*_*ef_PED*_ = 1/2Δ*f*_*PED*_) were also computed following the same approach as for inbreeding. Rates of change in genomic homozygosity (Δ*H*_*SNP*(*y*)_ and Δ*H*_*SNP*_) and similarity (Δ*S*_*SNP*(*y*)_ and Δ*S*_*SNP*_), and the corresponding estimates of *N*_*e*_ (*N*_*eH_SNP*_ = 1/2Δ*H*_*SNP*_, and *N*_*eS_SNP*_ = 1/2Δ*S*_*SNP*_) were also obtained.

## Results

Table 1 shows the mean, range, variance and coefficient of variation for the genomic homozygosity, similarity, inbreeding and coancestry coefficients. As expected, the molecular homozygosity and similarity coefficients were much higher than the inbreeding and coancestry coefficients. The reason is that the former (that are obtained on a SNP-by-SNP basis), do not discriminate alleles that are IBD or IBS. Estimates of *F* and *f*, based on ROHs and IBD segments, respectively, were close to the pedigree-based coefficients, indicating that they reflect well IBD. The highest coefficients of variation were observed for pedigree-based estimates.

**Table 1:** Mean, range (minimum and maximum values), variance and coefficient of variation (CV) for different estimates.

	Mean	Range	Variance	CV
*F*_*ROH*_	0.0770	0.0000–0.2660	0.0010	0.4125
*F*_*PED*_	0.0422	0.0000–0.2786	0.0007	0.6316
*H*_*SNP*_	0.6451	0.4890–0.8769	0.0002	0.0206
*f*_*SEG*_	0.0780	0.0000–0.5712	0.0006	0.3106
*f*_*PED*_	0.0439	0.0000–0.4180	0.0005	0.5173
*S*_*SNP*_	0.6447	0.5474–0.8366	0.0001	0.0148

*F*_*ROH*_: inbreeding based on ROHs; *F*_*PED*_: pedigree-based inbreeding; *H*_*SNP*_: SNP-by-SNP-based homozygosity; *f*_*SEG*_: coancestry based on IBD segments; *f*_*PED*_: pedigree-based coancestry; *S*_*SNP*_: SNP-by-SNP-based similarity

Table 2 shows the relationships between the genomic homozygosity and similarity, and different inbreeding and coancestry coefficients. The correlation between *F*_*ROH*_ and *F*_*PED*_ was higher than that between *H*_*SNP*_ and *F*_*PED*_ but still relatively low. Actually, 33% and 26% of the variance in *F*_*PED*_ can be explained by *F*_*ROH*_ and *H*_*SNP*_, respectively. The highest correlation was that between *H*_*SNP*_ and *F*_*ROH*_. Seventy seven percent of the variance in *F*_*ROH*_ can be explained by *H*_*SNP*_. Pearson’s correlation coefficients involving *S*_*SNP*_, *f*_*SEG*_ and *f*_*PED*_ were substantially higher than those involving *H*_*SNP*_, *F*_*ROH*_, *F*_*PED*_. In fact, 61% and 53% of the variance in *f*_*PED*_ is explained by *f*_*SEG*_ and *S*_*SNP*_, respectively. Also, 90% of the variance in *f*_*SEG*_ is explained by *S*_*SNP*_.

**Table 2:** Intercept (*a*), regression coefficient (*b*) and correlation (*R*) between different estimates.

Regression of			*a*	*b*	*R*
*F*_*PED*_	on	*F*_*ROH*_	0.01	0.48	0.57
*F*_*PED*_	on	*H*_*SNP*_	−0.62	1.03	0.51
*F*_*ROH*_	on	*H*_*SNP*_	−1.29	2.12	0.88
*f*_*PED*_	on	*f*_*SEG*_	−0.01	0.73	0.78
*f*_*PED*_	on	*S*_*SNP*_	−1.08	1.74	0.73
*f*_*SEG*_	on	*S*_*SNP*_	−1.49	2.43	0.95

*F*_*PED*_: pedigree-based inbreeding; *F*_*ROH*_: inbreeding based on ROHs; *H*_*SNP*_: SNP-by-SNP based homozygosity; *f*_*PED*_: pedigree-based coancestry; *f*_*SEG*_: coancestry based on IBD segments; *S*_*SNP*_: SNP-by-SNP based similarity

Table 3 shows the rates of change in genomic homozygosity and similarity and in genomic and pedigree-based inbreeding and coancestry per year and per generation, and the *N*_*e*_ estimated from those rates. Despite the different scales of the SNP-by-SNP based measures when compared with the inbreeding and coancestry coefficients (see Table 1), the rates of change were very similar for all these parameters, as expected. Consequently, estimates of *N*_*e*_ obtained from Δ*S*_*SNP*_ and from Δ*f*_*SEG*_ and Δ*f*_*PED*_ were very close. Rates of change in *H*_*SNP*_, *F*_*SEG*_ and *F*_*PED*_ were also very close. Pedigree-based inbreeding rates were slightly lower than the corresponding coancestry rates. Similarly, rates of genomic homozygosity were slightly lower than the corresponding rates of genomic similarity. Consequently, estimates of *N*_*e*_ obtained from rates of change in molecular homozygosity and inbreeding were slightly higher than estimates obtained from rates of change in molecular similarity and coancestry.

**Table 3:** Rates of change in inbreeding, molecular homozygosity, coancestry and molecular similarity per year (Δ*F*_(y)_, Δ*H*_(y)_, Δ*f*_(y)_ and Δ*S*_(y)_), respectively, and per generation (Δ*F*, Δ*H*, Δ*f* and Δ*S*) using different sources of information, and estimates of effective population sizes obtained from Δ*F* (*N_e_*_*F*_), Δ*H* (*N_e_*_*H*_), Δ*f* (*N_e_*_*f*_) and from Δ*S* (*N_e_*_*S*_).

	ROHs or IBD segments	SNP-by-SNP	Pedigree
Δ*F*_(*y*)_ or Δ*H*_(*y*)_	0.0016	0.0016	0.0015
Δ*F* or Δ*H*	0.0067	0.0067	0.0063
*N*_*eF*_ or *N*_*eH*_	74.4	74.4	79.4
Δ*f*_(*y*)_ or Δ*S*_(*y*)_	0.0016	0.0017	0.0018
Δ*f* or Δ*S*	0.0067	0.0071	0.0076
*N*_*ef*_ or *N*_*eS*_	74.4	70.0	66.1

Given that *L* decreased over time, we also estimated *N*_*e*_ for different periods (1960 to 1979, 1980 to 1999 and 2000 to 2013). Annual rates of homozygosity and inbreeding remained constant across time, but *N*_*e*_ increased as a consequence of the reduction in *L*. These estimates of *N*_*e*_ ranged from 61.3 to 65.4 for the period from 1960 to 1979, from 74.4 to 79.4 for the period from 1980 to 1999, and from 94.7 to 101.0 for the period from 2000 to 2013.

## Discussion

In this study, estimates of coancestry, inbreeding and *N*_*e*_ obtained from genome-wide information have been compared with those obtained from pedigree information in the Spanish population of Holstein cattle. Our results confirm the small *N*_*e*_ of the Holstein breed and the need of controlling coancestry and inbreeding rates.

The correlation between *F*_*ROH*_ and *F*_*PED*_ (0.57) obtained in the present study is in the range of values obtained in previous studies. Ferenčaković et al. [22] found values ranging from 0.50 to 0.72 when analysing four different cattle breeds with the 50 K chip. Keller et al. [20] showed that *F*_*ROH*_ is preferable to *F*_*PED*_ and other measures of genomic inbreeding because it correlates better with inbreeding depression. More specifically, for a *N*_*e*_ similar to that estimated here for the Holstein population, they predicted that the correlation between homozygous mutation load and inbreeding coefficient was higher for *F*_*ROH*_ (0.60) than for *F*_*PED*_ (0.25).

Molecular homozygosity and similarity coefficients calculated on a SNP-by-SNP basis were much higher than pedigree-based inbreeding and coancestry coefficients because the latter refer to a base population where no homozygosity exists. Thus, with the SNP-by-SNP based method alleles that are IBD and IBS can not be distinguished. Several approaches have been proposed to express these genomic coefficients in the same scale as pedigree-based coefficients [37] but they require knowing the base population allele frequencies. However, given that these frequencies are usually unknown, these methods are generally unaccurate [38, 39]. Another reason for correcting the raw molecular homozygosity and / or similarity is that in genome-wide evaluation methods, genomic relationship matrices are usually combined with pedigree-based relationship matrices because SNP genotypes are not available for many animals included in the evaluation procedure [21, 40, 41, 42]. Genomic matrices are usually corrected using the frequencies estimated in the present population through the formula of VanRaden [43, 44]. An alternative would be to use genomic matrices based on IBD segments rather that are in the same scale as pedigree-based matrices. This could represent an advantage over current approaches used in the context of genome-wide selection for predicting breeding values [45].

Here, estimates of molecular homozygosity, molecular similarity, genomic inbreeding and coancestry were obtained using genotypes contained in the Illumina BovineSNP50 BeadChip. A higher density cattle chip (i.e., the BovineHD BeadChip) containing 777,962 SNPs is also commercially available and used in dairy cattle genetic evaluations. Although in principle it is expected that increasing marker density would lead to an increase in the accuracy of genomic predictions, there are indications that this is not the case at least when *N*_*e*_ is low [46]. Low *N*_*e*_ is associated with high linkage disequilibrium and, thus, increasing marker density above a certain level may not improve significantly the accuracy of genomic evaluations [47]. In fact, small differences have been detected in genomic prediction when comparing the high density panel with the 50 K chip [48]. In a similar way, two different studies have showed that the use of the high density chip does not lead to higher accuracies when estimating inbreeding [23, 49]. For three different cattle breeds, Ferenčaković et al. [23] found similar correlations between *F*_*ROH*_ and *F*_*PED*_ when using the 50 K chip (estimates ranging from 0.62 to 0.77) and when using the high density cattle panel (estimates ranging from 0.61 to 0.75). Also, Purfield et al. [49] analysed nine different cattle breeds and indicated that the percentages of the variance in *F*_*PED*_ explained by *F*_*ROH*_ with the high density and 50 k chips were very similar (56% and 53%, respectively).

As expected, rates of change in molecular homozygosity, and genomic and pedigree-based inbreeding (and rates of change in molecular similarity, and genomic and pedigree-based coancestry) were very similar [26, 38]. Rates of change were slightly lower for molecular homozygosity and inbreeding than for molecular similarity and coancestry, and consequently, *N*_*eH*_ and *N*_*eF*_ estimates were slightly higher than *N*_*eS*_ and *N*_*ef*_ estimates. This result could be a reflection of non-random mating (i. e. avoidance of matings between relatives) if the level of non-randomness was not constant across generations [50].

For cattle, there is evidence that the levels of *N*_*e*_ were large (of the order of tens of thousands or more) following domestication [2, 3], but current *N*_*e*_ in some modern breeds are of the order of 100 [2, 3]. Estimates of *N*_*e*_ obtained in this study across different time periods are close to those previously published for other Holstein populations. Estimates of *N*_*e*_ obtained from pedigree data have ranged from 39 in the US population [51] to 115 in the Canadian population [52]. Estimates obtained from genome-wide data have ranged from around 80 in the US population [53] to 150 in the Australian Holstein cattle [54].

The small *N*_*e*_ found in Holstein cattle reflects the fact that breeding strategies followed in this breed have implied a very heavy use of few top sires and reinforces the need of controlling the rate at which coancestry and inbreeding increase in selection programmes [2]. Both selection and mating strategies have been proposed in the past for controlling the rates of coancestry and inbreeding. In particular, optimum contribution selection [55, 56] provides a useful tool to manage the rate of accumulation of inbreeding. When applying this selection tool in cattle, higher genetic gains are expected at a given rate of inbreeding [4]. Both objectives can be attained using programmes where selection is based on classical BLUP-EBVs and those based on genomic EBVs [19]. The application of the method in Holstein cattle would however require a coordinated policy on the use of selected candidates and on breeding objectives [4]. This may require the cooperation of breeders and artificial insemination organizations to ensure that the correct animals and their contributions are identified [57, 58]. Recently, Pryce et al. [28] and Sun et al. [59] have showed that mating strategies based on genomic information can reduce progeny inbreeding when compared with strategies based on pedigree information. Thus, dense genetic marker information is not only valuable for increasing accuracies of selection but also for controlling the rate at which coancestry and inbreeding increase in dairy cattle breeding programmes [19].

## References

[1] Falconer DS, Mackay TFC (1996) Introduction to quantitative genetics 4th edition UK: Longman, Harlow.

[2] Brotherstone S, Goddard M (2005) Artificial selection and maintenance of genetic variance in the global dairy cow population. Philos Trans R Soc Lond B Biol Sci 360: 1479–1488. 16048790 10.1098/rstb.2005.1668 PMC1569519

[3] Hill WG (2010) Understanding and using quantitative genetic variation. Philos Trans R Soc Lond B Biol Sci 365: 73–85. 10.1098/rstb.2009.0203 20008387 PMC2842708

[4] Kearney JF, Wall E, Villanueva B, Coffey MP (2004) Inbreeding trends and application of optimized selection in the UK Holstein population. J Dairy Sci 87: 3503–3509. 15377628 10.3168/jds.S0022-0302(04)73485-2

[5] González-Recio O, López de Maturana E, Gutiérrez JP (2007) Inbreeding depression on female fertility and calving ease in Spanish dairy cattle. J Dairy Sci 90: 5744–5752. 18024768 10.3168/jds.2007-0203

[6] Parland SM, Kearney JF, Rath M, Berry DP (2007) Inbreeding effects on milk production, calving performance, fertility and conformation in Irish Holstein-Friesians. J Dairy Sci 90: 4411–4419. 17699061 10.3168/jds.2007-0227

[7] Miglior F, Van Doormaal BJ, Kistemaker G (2008) Phenotypic analysis of inbreeding depression for traits measured in Canadian dairy cattle breeds. http://www.cdn.ca/Articles/GEBMAY2008/Industry_Session_Agenda-May2008.pdf.

[8] Bjelland DW, Weigel KA, Vukasinovic N, Nkrumah JD (2013) Evaluation of inbreeding depression in Holstein cattle using whole-genome SNP markers and alternative measures of genomic inbreeding. J Dairy Sci 96: 4697–4706. 10.3168/jds.2012-6435 23684028

[9] Schütz E, Scharfenstein M, Brenig B (2008) Implication of complex vertebral malformation and bovine leukocyte adhesion deficiency DNA-based testing on disease frequency in the Holstein population. J Dairy Sci 91: 4854–4859. 10.3168/jds.2008-1154 19038961

[10] Shanks RD, Dombrowski DB, Harpestad GW, Robinson JL (1984) Inheritance of UMP synthase in dairy cattle. J Hered 75: 337–340. 6548235 10.1093/oxfordjournals.jhered.a109951

[11] Charlier C, Agerholm JS, Coppieters W, Karlskov-Mortensen P, Li W, de Jorn G, et al (2012) A deletion in the bovine *FANCI* gene compromises fertility by causing fetal death and brachyspina. PLoS ONE 7(8): e43085 10.1371/journal.pone.0043085 22952632 PMC3430679

[12] Kehrli ME Jr, Schmalstieg FC, Anderson DC, Van der Maaten MJ, Hughes BJ, Ackermann MR, et al (1990) Molecular definition of the bovine granulocytopathy syndrome: Identification of deficiency of the Mac-1 (CD11b/CD18) glycoprotein. Am J Vet Res 51: 1826–1836. 1978618

[13] Meuwissen TH, Hayes BJ, Goddard ME (2001) Prediction of total genetic value using genome-wide dense marker maps. Genetics 157: 1819–1829. 11290733 10.1093/genetics/157.4.1819 PMC1461589

[14] Lund M, de Ross APW, de Vries A, Druet T, Ducrocq V, Fritz S, et al (2011) A common reference population from four European Holstein populations increases reliability of genomic predictions. Genet Sel Evol 43: 43 10.1186/1297-9686-43-43 22152008 PMC3292506

[15] VanRaden PM, Cooper T (2011) The genomic evaluation system in the United States: Past, present, future. J Dairy Sci 94: 3202–3211. 10.3168/jds.2010-3866 21605789

[16] Schaeffer LR (2006) Strategy for applying genome-wide selection in dairy cattle. J Anim Breed Genet 123: 218–223. 16882088 10.1111/j.1439-0388.2006.00595.x

[17] Daetwyler HD, Villanueva B, Bijma P, Woolliams JA (2007) Inbreeding in genome-wide selection. J Anim Breed Genet 124: 369–376. 18076474 10.1111/j.1439-0388.2007.00693.x

[18] de Roos APW, Schrooten C, Veerkamp RF, van Arendonk JAM (2011) Effects of genomic selection on genetic improvement, inbreeding, and merit of young versus proven bulls. J Dairy Sci 94: 1559–1567. 10.3168/jds.2010-3354 21338821

[19] Sonesson AK, Woolliams JA, Meuwissen TH (2012) Genomic selection requires genomic control of inbreeding. Genet Sel Evol 44: 27 10.1186/1297-9686-44-27 22898324 PMC3522025

[20] Keller MC, Visscher PM, Goddard ME (2011) Quantification of inbreeding due to distant ancestors and its detection using dense single nucleotide polymorphism data. Genetics 189: 237–249. 10.1534/genetics.111.130922 21705750 PMC3176119

[21] VanRaden PM, Olson KM, Wiggans GR, Cole JB, Tooker ME (2011) Genomic inbreeding and relationships among Holstein, Jersey, and Brown Swiss. J Dairy Sci 94: 5673–5682. 10.3168/jds.2011-4500 22032391

[22] Ferenčaković M, Hamzić E, Gredler B, Solberg TR, Klemetsdal G, Curik I, et al (2012) Estimates of autozygosity derived from runs of homozygosity: empirical evidence from selected cattle populations. J Anim Breed Genet 130: 286–293. 10.1111/jbg.12012 23855630

[23] Ferenčaković M, Sölkner J, Curik I (2013) Estimating autozygosity from high-throughput information: effects of SNP density and genotyping errors. Genet Sel Evol 45: 42 10.1186/1297-9686-45-42 24168655 PMC4176748

[24] Gómez-Romano F, Sölkner J, Villanueva B, Mészáros G, de Cara MAR, Pérez O'Brien AM, et al. (2014) Genomic estimates of inbreeding and coancestry in Brown Swiss cattle. Proceedings of the 10th World Congress of Genetics Applied to Livestock Production. 17–22 August, Vancouver, Canada.

[25] Li M-H, Strandén I, Tiirikka T, Sevón-Aimonen M-L, Kantanen J (2011) A comparison of approaches to estimate the inbreeding coefficient and pairwise relatedness using genomic and pedigree data in a sheep population. PLoS ONE 6(11): e26256 10.1371/journal.pone.0026256 22114661 PMC3220595

[26] Saura M, Fernández A, Rodríguez MC, Toro MA, Barragán C, Fernández AI, et al (2013) Genome-wide estimates of coancestry and inbreeding in a closed herd of ancient Iberian pigs. PLoS ONE 8(10): e78314 10.1371/journal.pone.0078314 24205195 PMC3814548

[27] Silió L, Rodríguez MC, Fernández A, Barragán C, Benítez R, Óvilo C, et al (2013) Measuring inbreeding and inbreeding depression on pig growth from pedigree or SNP-derived metrics. J Anim Breed Genet 130: 349–360. 10.1111/jbg.12031 24074172

[28] Pryce JE, Hayes BJ, Goddard ME (2012) Novel strategies to minimize progeny inbreeding while maximizing genetic gain using genomic information. J Dairy Sci 95: 377–388. 10.3168/jds.2011-4254 22192217

[29] Toro MA, Villanueva B, Fernández J (2014) Genomics and conservation genetics. Livest Sci 166: 48–53.

[30] Zimin AV, Delcher AL, Florea L, Kelley DR, Schatz MC, Puiu D, et al (2009) A whole-genome assembly of the domestic cow, *Bos taurus* . Genome Biol 10: R42 10.1186/gb-2009-10-4-r42 19393038 PMC2688933

[31] Gutiérrez JP, Goyache F (2005) A note on ENDOG: a computer program for analysing pedigree information. J Anim Breed Genet 122: 172–176. 16130468 10.1111/j.1439-0388.2005.00512.x

[32] de Cara MAR, Villanueva B, Toro MA, Fernández J (2013) Using genomic tools to maintain diversity and fitness in conservation programmes. Mol Ecol 22: 6091–6099. 10.1111/mec.12560 24128280

[33] Browning SR, Browning BL (2007) Rapid and accurate haplotype phasing and missing data inference for whole genome association studies using localized haplotype clustering. Am J Hum Genet 81: 1084–1097. 17924348 10.1086/521987 PMC2265661

[34] Malécot G (1948) Les mathématiques de l’hérédité Paris: Masson & Cie.

[35] Boichard D (2002) PEDIG: A Fortran package for pedigree analysis suited for large populations. Proc 7th World Cong Genet Appl Livest Prod, Montpellier, France. CD-ROM communication no. 28–13.

[36] Meuwissen TH, Luo Z (1992) Computing inbreeding coefficients in large populations. Genet Sel Evol 24: 305–313.

[37] Toro MA, García-Cortés LA, Legarra A (2011) A note on the rationale for estimating genealogical coancestry from molecular markers. Genet Sel Evol 43: 27 10.1186/1297-9686-43-27 21749687 PMC3154857

[38] Toro MA, Barragán C, Óvilo C, Rodrigáñez J, Rodríguez C, Silió L (2002) Estimation of co-ancestry in Iberian pigs using molecular markers. Conserv Genet 3: 309–320.

[39] Rodríguez-Ramilo ST, Toro MA, Martínez P, Castro J, Bouza C, Fernández J (2007). Accuracy of pairwise methods in the reconstruction of family relationships, using molecular information from turbot (*Scophthalmus maximus*). Aquaculture 273: 434–442.

[40] Forni S, Aguilar I, Misztal I (2011) Different genomic relationship matrices for single-step analysis using phenotypic, pedigree and genomic information. Genet Sel Evol 43: 1 10.1186/1297-9686-43-27 21208445 PMC3022661

[41] Meuwissen TH, Luan T, Woolliams JA (2011) The unified approach to the use of genomic and pedigree information in genomic evaluations revisited. J Anim Breed Genet 128: 429–439. 10.1111/j.1439-0388.2011.00966.x 22059576

[42] Rodríguez-Ramilo ST, García-Cortés LA, González-Recio Ó (2014) Combining genomic and genealogical information in a Reproducing Kernel Hilbert Spaces regression model for genome-enabled predictions in dairy cattle. PLoS ONE 9(3): e93424 10.1371/journal.pone.0093424 24671175 PMC3966896

[43] VanRaden PM (2007) Genomic measures of relationship and inbreeding. Interbull Bull 37: 33–36.

[44] VanRaden PM (2008) Efficient methods to compute genomic predictions. J Dairy Sci 91: 4414–4423. 10.3168/jds.2007-0980 18946147

[45] Luan T, Yu X, Dolezal M, Bagnato A, Meuwissen THE (2014) Genomic prediction based on runs of homozygosity. Genet Sel Evol 46: 64 25284459 10.1186/s12711-014-0064-6 PMC4189176

[46] MacLeod IM, Hayes BJ, Goddard ME (2013) Will sequence SNP data improve the accuracy of genomic prediction in the presence of long term selection? Proc Asscoc Advmt Anim Breed Genet 20: 215–219.

[47] Gómez-Romano F, Villanueva B, de Cara MAR, Fernández J (2013) Maintaining genetic diversity using molecular coancestry: the effect of marker density and effective population size. Genet Sel Evol 45: 38 10.1186/1297-9686-45-38 24088414 PMC3852135

[48] Su G, Brøndum RF, Ma P, Guldbrandtsen B, Aamand GP, Lund MS (2012) Comparison of genomic predictions using medium-density (54000) and high-density (777000) single nucleotide polymorphism marker panels in Nordic Holstein and Red Dairy Cattle populations. J Dairy Sci 95: 4657–4665. 10.3168/jds.2012-5379 22818480

[49] Purfield DC, Berry DP, Mc Parland S, Bradley DG (2012) Runs of homozygosity and population history in cattle. BMC Genet 13: 70 10.1186/1471-2156-13-70 22888858 PMC3502433

[50] Caballero A (1994) Developments in the prediction of effective population size. Heredity 73: 657–679. 7814264 10.1038/hdy.1994.174

[51] Weigel KA (2001) Controlling inbreeding in modern breeding programs. J Dairy Sci 84: E177–E184.

[52] Stachowicz K, Sargolzaei M, Miglior F, Schenkel FS (2011) Rates of inbreeding and genetic diversity in Canadian Holstein and Jersey cattle. J Dairy Sci 94: 5160–5175. 10.3168/jds.2010-3308 21943766

[53] Sargolzaei M, Schenkel FS, Jansen GB, Schaeffer LR (2008) Extent of linkage disequlibrium in Holstein cattle in North America. J Dairy Sci 91: 2106–2117. 10.3168/jds.2007-0553 18420642

[54] Hayes BJ, Visscher PM, McPartlan HC, Goddard ME (2003) Novel multilocus measure of linkage disequilibrium to estimate past effective population size. Genome Res 13: 635–643. 12654718 10.1101/gr.387103 PMC430161

[55] Meuwissen TH (1997) Maximizing the response of selection with a predefined rate of inbreeding. J Anim Sci 75:934–940. 9110204 10.2527/1997.754934x

[56] Grundy B, Villanueva B, Woolliams JA (1998) Dynamic selection procedures for constrained inbreeding and their consequences for pedigree development. Genet Res 72:159–168.

[57] Weigel KA, Lin SW (2002) Controlling inbreeding by constraining the average relationship between parents of young bulls entering AI progeny test programs. J Dairy Sci 85: 2376–2383. 12362471 10.3168/jds.s0022-0302(02)74318-x

[58] Avendaño SA, Villanueva B, Woolliams JA (2003) Expected increases in genetic merit from using optimized contributions in two livestock populations of beef cattle and sheep. J Anim Sci 81: 2964–2975. 14677851 10.2527/2003.81122964x

[59] Sun C, VanRaden PM, O’Connell JR, Weigel KA, Gianola D (2013) Mating programs including genomic relationships and dominance effects. J Dairy Sci 96: 8014–8023. 10.3168/jds.2013-6969 24119810

